# Cross-cultural adaptation and psychometric evaluation of the “Modification of Hall’s professionalism scale for use with pharmacists”

**DOI:** 10.1186/s12909-023-04815-y

**Published:** 2023-11-16

**Authors:** Fernando de Castro Araújo Neto, Thaís Maria Araújo Tavares, Douglas de Menezes Santos, Francielly Lima da Fonseca, Dyego Carlos Souza Anacleto de Araújo, Alessandra Rezende Mesquita, Divaldo Pereira de Lyra

**Affiliations:** 1 Laboratory of Teaching and Research in Social Pharmacy, Department of Pharmacy, Federal University of Sergipe - São Cristóvão Campus, São Cristóvão - Sergipe, Brazil; 2 Health Sciences Graduate Program, University Hospital of Sergipe - Federal University of Sergipe, Aracaju Campus, Aracaju - Sergipe, Brazil; 3 Pharmaceutical Science Graduate Program, Federal University of Sergipe, São Cristóvão Campus, São Cristóvão - Sergipe, Brazil; 4 Laboratory of Innovation in Pharmaceutical Care, Department of Pharmaceutical Sciences, Federal University of Espírito Santo - Maruípe Campus, Vitória - Espírito Santo, Brazil

**Keywords:** Professionalism, Pharmacy, Pharmacists, Instrument, Validation, Cross-cultural adaptation

## Abstract

**Background:**

Professionalism is the demonstration of behaviors that guide the actions of health professionals. In Pharmacy, its implementation is possible through assessment instruments for pharmacists, such as the “Modification of Hall’s Professionalism Scale for Use with Pharmacists”.

**Objective:**

To translate the “Modification of Hall’s Professionalism Scale for Use with Pharmacists” into a Brazilian Portuguese version and evaluate its psychometric properties for pharmacists.

**Method:**

The methodological process of this study took place in three stages: translation and cross-cultural adaptation of the instrument original version into a Brazilian Portuguese version; validation of the scale content through consensus among geographically distinct experts and, finally; examination of the scale psychometric measurement properties through a convenience sample of 600 Brazilian pharmacists. At this stage, construct validity was verified using Exploratory Factor Analysis (EFA) and reliability was examined by calculating the composite reliability.

**Results:**

The adapted instrument to a Brazilian Portuguese version demonstrated content validity with coefficients considered acceptable, above 0.8. The EFA demonstrated a structure supported by six factors and 39 items. The H index suggested high stability for all factors as well as composite reliability.

**Conclusion:**

The Brazilian Portuguese version of the instrument presented appropriate content validity coefficients and psychometric properties. This measure may be useful for future studies on professionalism regarding teaching strategies and assessment of this construct among pharmacists.

**Supplementary Information:**

The online version contains supplementary material available at 10.1186/s12909-023-04815-y.

## Introduction

Professionalism is the expression of behaviors and attitudes that guide professions. That is, a social contract signed between professionals and society [1–3]. Although this definition is not a universal consensus, it arises from the pragmatic changes that health professions have undergone to develop patient-centered models of practice. In recent years, this has reflected in the increased interest in the topic of professionalism, even as a comprehensive construct or as an autonomous competence, substantially within the scope of research in social pharmacy [1, 3–7].

In this context, Pharmacy has gone through transitions motivated by questions about its ethics and autonomy in the face of dilemmas related to the profit obtained from the medicine trade. This aspect is commonly dissociated from patient care services [4, 5, 8, 9]. With this, it is clear the need to operationalize professionalism, that is, the proposition of tools to enable its understanding by students and pharmacists, through teaching and accomplishment of changes in the work processes in line with social demands [10–13].

Professionalism is also understood as a variable to be measured and evaluated through instruments that systematize perceptions on the subject, from the transformation of behaviors and values, such as altruism and autonomy, into symbols of what Pharmacy expects pharmacists to perform in different scenarios [1, 3, 14]. In order to reinforce the demand, the literature recommends that these measures must have evidence of their validity and reliability, which suggests the harmonic interpretation of their results and reproducibility [3].

To enable the instrumentalization of professionalism as a measurable phenomenon in Pharmacy, the literature presents the instrument “Modification of Hall’s Professionalism Scale for Use with Pharmacists”, developed by Schack and Hepler (1979). To this extent, professionalism is treated as a facet that encompasses behaviors considered “professional”, based on the proposition of situations and the judgment of the pharmacist’s agreement with each one, in a process described in literature as reflection and self-assessment [15].

With evidence of its reliability, this instrument has been used in some countries, especially in the United States [16, 17]. This reinforces the need for studies that verify its use among pharmacists from other cultures. Moreover, in Brazil, the changes in the work process for patient care aligned to the interest on professionalism are on the rise [4, 9, 18–21]. Therefore, the aim of this study was to translate the “Modification of Hall’s Professionalism Scale for Use with Pharmacists” into Brazilian Portuguese language and evaluate its psychometric properties for pharmacists.

## Methods

The methodological process was developed in three steps: 1) translation and cross-cultural adaptation; 2) assessment of content-based validity evidence; and 3) assessment of psychometric properties. The instrument “Modification of Hall’s Professionalism Scale for Use with Pharmacists” was developed by Charles Hepler, pharmacist and reference regarding the philosophy and model of professional practice for pharmacists. This scale was developed based on Richard Hall's proposal to assess the professionalism of physicians, lawyers, nurses, and other liberal professionals [22, 23].

The instrument has 40 statements that are evaluated using a five-point Likert scale, ranging from “totally disagree” to “totally agree”. The statements in this instrument are grouped into six domains: autonomy, vocation, professional council (in Brazil, the Federal Council of Pharmacy) as the main reference, self-regulation, continuing education, and altruism. This instrument has another version, in Thai language [17]. The theoretical framework adopted in the conception of this instrument factors is included in the Supplementary material.

### Translation and cross-cultural adaptation of the “Modification of Hall’s Professionalism Scale for Use with Pharmacists” into Brazilian Portuguese language

The process of translation and cross-cultural adaptation of the “Modification of Hall’s Professionalism Scale for Use with Pharmacists” into Brazilian Portuguese language was authorized by the corresponding author and followed the recommendations of Borsa, Damásio, and Bandeira, 2012. This process was developed in six stages: translation, translation synthesis, experts review, target audience evaluation, and back-translation [24, 25].

The translation was carried out independently by two bilingual translators, both Brazilian Portuguese native speakers and fluent in English [25]. One of the translators was aware of the objectives and the theoretical framework surrounding the instrument, while the other “native translator” was not [26]. The two translations (T1 and T2) were compared by the researchers and disagreements were resolved by consensus, originating the T12 version [27].

A panel of experts, composed of an English teacher, three experts in validation studies and the researchers responsible for the study analyzed the translations (T1 and T2), the synthesis (T12) and the original version, in order to assess whether the translated and adapted version maintained the following equivalences [24, 26, 28]:

- **Semantic equivalence:** if the words had the same meaning, if there was any ambiguity in the words or grammatical errors.
- **Idiomatic equivalence:** whether the translated items were adapted using expressions that maintain the cultural meaning of the item.
- **Experiential equivalence:** whether the translated item was culturally applicable, or its replacement would be necessary.
- **Conceptual equivalence:** whether the item, even translated, was faithful to the meaning when evaluating the same aspects in two different cultures or languages.

After the discussions and changes proposed by the panel of experts, the T3 version was obtained, and submitted to the target audience of the instrument for evaluation. The target audience, composed of nine geographically distant pharmacists, qualitatively evaluated the statements and reported whether they experienced any difficulty in understanding the items [28].

After the changes proposed by the target audience, this version went to the back-translation stage. The back-translation was performed by two translators, both English native speakers and fluent in Portuguese that did not have any knowledge about the original version of the instrument. Thus, the RT1 and RT2 versions were originated [27]. The two versions were compared by the study researchers, ensuring the equivalence of the scales. The last version originated in this stage is included in the Supplementary material.

### Assessment of content-based validity evidence

The translated and adapted version of the “Modification of Hall’s Professionalism Scale for Use with Pharmacists” was submitted to evaluation of content-based validity evidence using the Delphi technique. To compose the expert panel responsible for the evaluation, a database search for professional curricula was carried out. To be considered an expert, the participant should have scored at least five points in the adapted Fehring’s Criteria (1987) [29, 30].

The invitation to compose the expert panel in this stage was sent via email to pharmacists across the country. These guests, whose curriculum was evaluated according to Fehring's Criteria, could indicate other experts to compose the panel. Following the literature recommendations, for each Delphi round it was required the participation of six professionals. The invitation was sent to ten experts and was accepted by six of them [28–30]. The Fehring’s Criteria for the composed expert panel and the characteristics of the experts who composed the Delphi round are available in the Supplementary material.

The Brazilian Portuguese instrument version was made available in electronic form through the Google Forms virtual platform (Google Inc, Mountain View, CA, USA). When accessing it, the experts were instructed to fill in the requested data and invited to check the data confidentiality and the free and informed consent terms [29]. Then, the experts anonymously and independently evaluated the items according to the five-point Likert scale that ranged from “totally disagree” to “totally agree”, considering the following criteria: [28, 31].

- **Clarity of language:** the language used in the construct was understandable by the target population and adequate for the purpose of the study.
- **Practical pertinence:** whether the assessed item was appropriate for the target population.
- **Theoretical relevance:** whether the item represented what it was intended to measure regarding to the theoretical framework used in the instrument proposal.

According to this judgment, it was offered a time for criticism and suggestions about the content. As recommended by the literature, at the end of each Delphi round, it was considered an agreement percentage of 80% using the Coefficient of Validity (CVC) to indicate consensus among experts and validation of the items. After this trial, the modifications proceeded to a second round [30].

### Assessment of psychometric properties

To assess the psychometric properties of the scale, the instrument was made available online through Google Forms for pharmacists throughout Brazil. As an inclusion criterion, professionals should have carried out their activities in the areas of hospital pharmacy, community pharmacy, public pharmacy, or pharmaceutical office. Data collection took place between March and August 2022. Participants were recruited by disseminating the instrument through social networks and e-mail.

Following literature recommendation, the sample was calculated based on the need of four to ten individuals to answer each item. Thus, as the translated and cross-culturally adapted instrument version had 40 items, 400 pharmacists were needed to guarantee the minimum recommended sample. Furthermore, considering the 234,301 registered with the Brazilian Federal Pharmacy Council, it was considered that 384 pharmacists should respond to the survey, based on a confidence level of 95% and sampling error of 5% [32, 33]. Before responding the instrument, the participants accessed the free and informed consent form. For these participants, sociodemographic data were also collected, such as gender, age, geographic region in which they reside, type of institution in which they carry out professional activities (public or private), and the occupational area in which they currently work. The collected data were tabulated using the Microsoft Excel software and then proceeded to the evaluation of validity evidence based on the instrument internal structure through exploratory analysis.

### Evaluation of validity evidence based on the internal structure

To our knowledge, this is the first Brazilian Portuguese version of the “Modification of Hall’s Professionalism Scale for Use with Pharmacists”, which is now called Brazilian version of the “Modification of Hall’s Professionalism Scale for Use with Pharmacists”. Yet, it is the first study to examine the psychometric properties of this scale in Brazilian Portuguese language. Therefore, to identify the underlying factor structure, the Exploratory Factor Analysis (EFA) method was applied.

The EFA was conducted using the Factor software (version 10.9.02), and was implemented with a matrix and the “Robust Diagonally Weighted Least Squares” (RDWLS) extraction method [34]. The factorability of the matrix was verified using the Kaiser Meyer-Olkin criterion, which verifies the adequacy of the sample, where values above 0.80 are “excellent” and the Barlett sphericity test, responsible for evaluating the hypothesis the items may not correlate. For this hypothesis to be rejected as expected, it is desirable that *p* < 0.05 [35].

The number of instrument factors was determined using the Parallel Analysis technique with random permutation of the observed data and Robust Promin rotation [28, 36]. Residue distribution was evaluated using Weighted Root Mean Square Residual. According to this index, values < 1.0 are considered good. The factor structure adequacy procedure was developed using the chi-square (χ 2) per degrees of freedom (df) ratio (χ 2 /gl < 3). In addition, the following criteria were also adopted: Comparative Fit Index (CFI) ≥ 0.95 [37], goodness of fit index (GFI) ≥ 0.95, Non-Normed Fit Index (NNFI) ≥ 0.95, Root Mean Square Error of Approximation (RMSEA) ≤ 0.08, *p* > 0.05, and root mean square of residuals ≤ 0.8 [28, 38].

The quality of the factorial solution was evaluated based on the factor determinacy index (> 0.90) and expected percentage of true differences (> 90%) [34]. The verification of factors stability was carried out using the H index which scores from 0 to 1 how well items together represent a factor [34, 39, 40]. On this scale, values greater than 0.8 suggest stability. Finally, for items that were grouped into two factors, Pratt's Correlation and literature related to professionalism were used to cluster the item in the most related factor. As well, items that presented a factorial load lower than 0.3 were not considered valid [41–44].

### Reliability

**Fig. 1:**
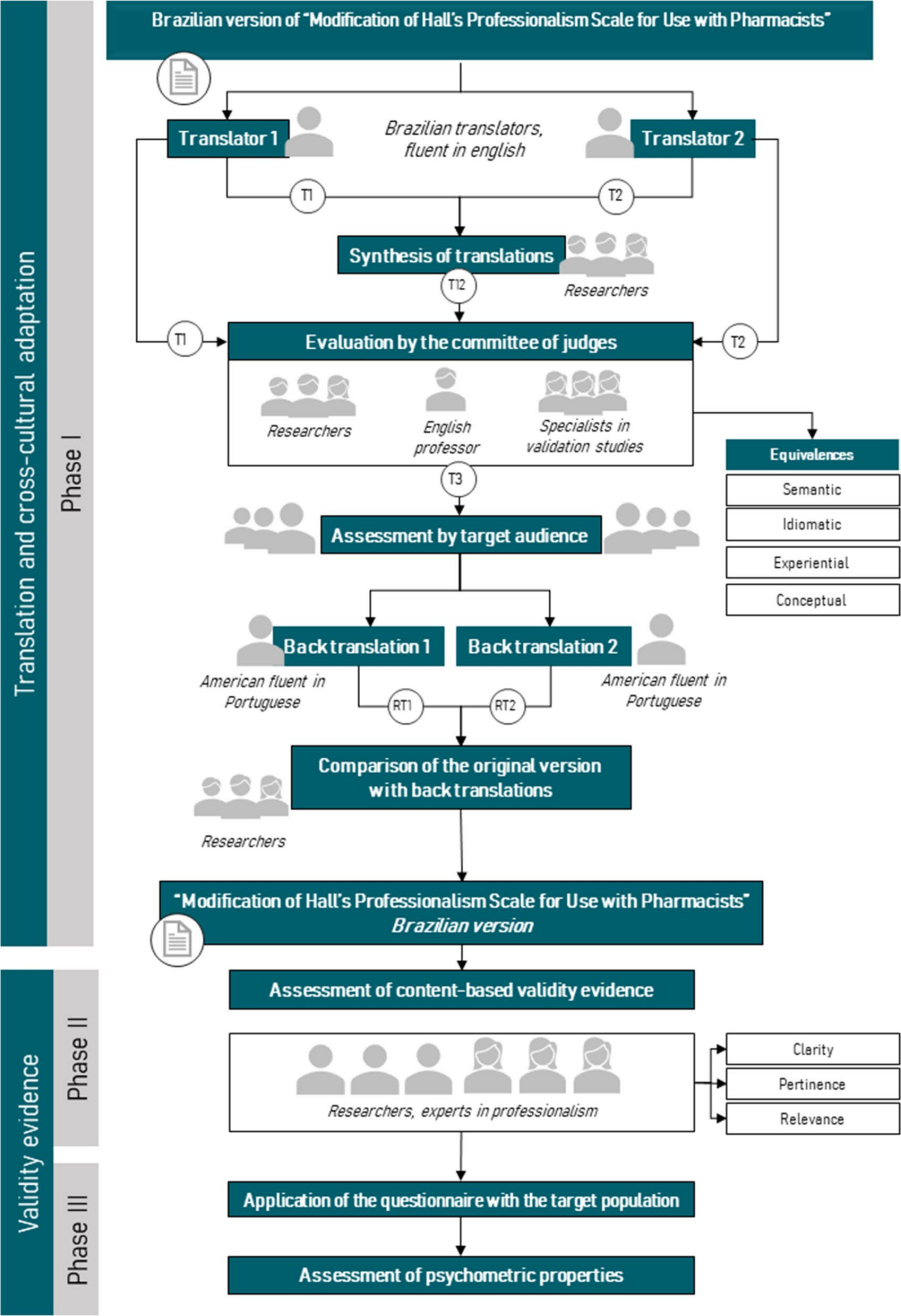
Study development stages. Source: prepared by the author

The reliability of the instrument was verified through composite reliability, which considers the magnitude of the factorial load of each item [28, 45]. Values ≥ 0.70, ≥ 0.80 and ≥ 0.90 indicate, respectively, acceptable, good and excellent internal consistency [46]. Figure 1 describes the study development stages.

### Ethical considerations

In compliance with the provisions of Resolution number 466/2012 of the National Health Council, this study was approved by the Research Ethics Committee of the Federal University of Sergipe under the project: Evaluation of Professionalism in the Pharmacy Area, Opinion No. 4169752.

## Results

### Assessment of content-based validity evidence

Six pharmacists from all geographic regions of Brazil participated in this stage and met the inclusion criteria based on Fehring's criteria for selection of judges, as well as the invitation. The minimum acceptable score was five. Table 1 below presents characteristics of the participants in the scale's content validation:

**Table 1:** Characteristics of the experts who were part of the committee of judges

Sex	Age	Academic degree	Professional experience	Score
Female	52 years	PhD	26 years	7 points
Female	47 years	PhD	16 years	7 points
Male	50 years	PhD	20 years	7points
Female	52 years	PhD	21 years	7 points
Female	35 years	PhD	9 years	7 points
Female	63 years	PhD	37 years	7 points

In the first round of evaluation of content-based validity evidence, the content validity coefficient ranged between 0.67 and 0.97. Items 1, 11, 18 and 23 did not reach the minimum score recommended by literature, which is 0.80 due to clarity of the statement. In addition to clarity, item 1 also did not reach the minimum coefficient due to pertinence and theoretical relevance. After adjusting the wording of items based on contributions provided by judges, the instrument went on to the second round of evaluation. In this stage, the items reached scores that varied between 0.88 and 0.92, which is considered validated. The data set referring to this stage is available in the Supplementary material of this manuscript.

### Assessment of psychometric properties

Six hundred pharmacists answered the instrument. Most of them were female (*n* = 415; 69.16%), lived in Northeastern Brazil (*n* = 228; 38%), and carried out their professional activities in private institutions (*n* = 335; 55.7%). Besides, community pharmacy was the area with the highest number of respondents (*n* = 299; 49.8%). The age of participants ranged from 21 to 72 years with an average of 34 years.

### Evaluation of validity evidence based on the internal structure

The results of Barlett's sphericity test (6737.0) and the KMO index (0.81) indicated the factorability of the item correlation matrix (Table 2). The parallel analysis suggested the retention of six factors, as well as in the original version of the instrument. The H index obtained for each factor suggested a high stability of the factorial structure. The other indices showed adequacy of the suggested model without the need for adjustments. Finally, the NNFI showed a 98% adjustment improvement, a result consistent with the other items. Table 2 presents validity evidence based on the instrument's internal structure:

**Table 2:** Validity evidence based on the scale's internal structure

**Exploratory Factor Analysis**		Adequacy of the correction matrix				Bartlett				6737.0 (df = 741)^a^	
						Kaiser–Meyer–Olkin (KMO)				0.81	
		Factors								6	
		Explained variance								53.7%	
		Waste distribution				Weighted Root Mean Square Residual (WRSR)				0.0356	
		Chi-square ratio for degree of freedom (X2/df)								757.625	
		Non-Normed Fit Index (NNFI)								0.98	
		Comparative Fit Index (CFI)								0.98	
		Goodness of Fit Index (GFI)								1.0	
		Root Mean Square Error of Approximation (RMSEA)								0.027	
		Root Mean Squared Residual (RSMR)								0.0437	
^a^*p* < 0,001; ^b^*p* = ,99											
Replicability adjusted by H index											
Factor Index Confidence Interval				Factor Index Confidence Interval				Factor Index Confidence Interval			
Autonomy				0.962				0.945 – 0.980			
Vocation				0.935				0.919 – 0.945			
Professional Council				0.878				0.799 – 0.915			
Self-regulation				0.838				0.795 – 0.858			
Continuing Education				0.867				0.810 – 0.887			
Altruism				0.828				0.779 – 0.845			
**Quality and effectiveness of factor score estimates**											
**Index**	**Factor**										
	**Autonomy**		**Vocation**		**Professional Council**		**Self-regulation**		**Continuing Education**		**Altruism**
**Factor Determinacy Index**	0.981		0.967		0.937		0.916		0.931		0.910
**Expected percentage of true differences**	96.2%		94.4%		91.5%		90%		91%		89.6%

The factorial loads of each item grouped into the respective factor can be observed in the table below. It is important to highlight that with low factor loading item 18, which presented factor loading -0.095, was removed, and the analytical procedure was redone to update the indices. The item presented low factor loadings on two other factors, these being "autonomy", with a factor loading of 0.26 and "self-regulation" with a factor loading of 0.21. Theoretically, during the literature analysis carried out by the researchers, none of these factors are related to the item, which in the original version of the Scale, is allocated to the “continuing education” factor. Therefore, in this version the item was removed from the final version of the Scale. Table 3 presents EFA results: items and factor loadings of the Brazilian version of the Modification of Hall’s Professionalism Scale for Use with Pharmacists.

**Table 3:**
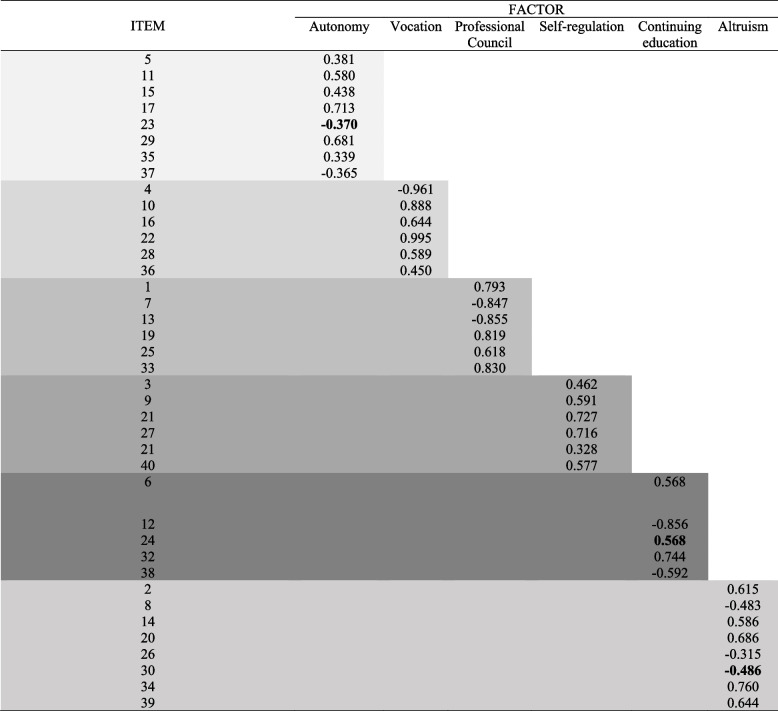
EFA results: items and factor loadings of the scale

### Reliability

The marker used in this study, composite reliability, showed values greater than 0.7 for each factor. Table 4 presents Reliability of the Brazilian version of the Modification of Hall’s Professionalism Scale for Use with Pharmacists.

**Table 4:** Reliability of the scale

Index	Factor					
	**Autonomy**	**Vocation**	**Professional Council**	**Self-regulation**	**Continuing Education**	**Altruism**
Composite reliability	0.715	0.898	0.912	0.745	0.803	0.800

## Discussion

In this study, the “Modification of Hall’s Professionalism Scale for Use with Pharmacists” was translated into Brazilian Portuguese language and adapted to Brazilian context. During this process, the translated version received suggestions for adjustments in its content by geographically distinct experts in addition to the translation procedures recommended by literature which generated a version with 40 items. After that, the instrument was submitted to the examination of psychometric properties through exploratory factor analysis, evaluation of replicability and reliability. This made it possible to present an instrument with evidence of validity based on content and internal structure with 39 items.

It is important to highlight that in this process, contrary to the original version of the instrument proposed by Schack e Hepler (1979) and the confirmatory analysis developed by Rupp and Segal (1989), all items underwent factor analysis, and in previous versions items 30, 31, 39 and 40 were conveniently ignored by the analysts. In this version, item 18, which in the original version represented the “continuing competence” factor, did not present a sufficient factorial load to group it into a domain, which led to its exclusion by the researchers. Thus, the current version has 39 items, grouped into six factors that can be answered using a five-item Likert scale ranging from “totally disagree” to “totally agree”.

The instrument exploratory factor analysis indicated a structure formed by six factors, and it is supported by the factor determinacy index and expected percentage of true differences. This indicates that the scale measures professionalism as a construct that reverberates in six factors. This structure was also shown in the original version of the instrument, proposed by Schack and Hepler (1979) and supported by the confirmatory analysis of Rupp and Segal (1989), conducted with 416 and 617 pharmacists, respectively. In this context, Rupp and Segal (1989) pointed the adequate representation of the model for the factors “autonomy” and “self-regulation” which, in this version, presented low factor loadings.

In the model proposed in the present study, the factor loadings of “autonomy” and “self-regulation” maintained acceptable indices. However, to assign items 24, 30, 31 and 37 to the factors “continuing education”, “altruism”, “self-regulation” and “autonomy”, the Pratt's correlation and literature were adopted as a reference. Other models of professionalism for pharmacists, pharmacy students and professions such as medicine have suggested structures that also vary between five and six factors [1, 13]. In literature, these factors are identified in other Pharmacy and Medicine instruments and associated with attitudes such as “responsibility” [1, 22, 47]. This corroborates the belief that there should be no lay interference during professional practice so that autonomy and control over one's own work are preserved.

This instrument showed excellent values in terms of replicability and reliability, in addition to high factorial stability. Similar results were identified by Schack and Hepler (1979), using Cronbach's alpha as a measure. In other version of this scale adapted to Thai language by Lerkiatbundit (2006), acceptable reliability was identified which suggests the reliability of the instrument for other languages than the original and in samples intended for non-American populations. In conventional scenarios, low values of these indexes would indicate the instability of the measure and, therefore, difficulties in its replicability [28, 34].

Regard to that, the present proposal aimed to promote discussions about professionalism in nations where this topic poorly arises such as Brazil, different from what traditionally happens in European countries and the United States. In fact, in Brazil, the Pharmacy work process has been currently modified to remove the stigma of an “incomplete profession” as it is apart from patients [1, 4, 48–51]. Thus, this instrument can be used to help pharmacists to better understand what they think about professionalism linked to the continuous process of changes in the philosophy and model of practice. It can also be useful to evaluate scenarios before and after the application of strategies for teaching professionalism or professional identity, and to support professional recruitment processes.

The present study has some strengths. To our knowledge, this is the first proposal for an instrument to assess pharmaceutical professionalism with evidence of validity based on content and internal structure for Brazilian Portuguese language. Furthermore, this instrument uses an original and consolidated measure in the social sciences, based on a cross-cultural adaptation process. Likewise, it presented favorable rates in accordance with literature, as the factorial analysis procedure replicated the original measure and incorporated items ignored before. As limitations, the high number of items can lead to fatigue or abandonment of respondents. Furthermore, in the EFA phase, the geographical concentration of interviewees in certain regions may have caused an over-representation of certain audiences. This may have been due to the fact that the sample came from a country with continental dimensions and cultural multiplicity. As future directions, other initiatives may address the reduction of scale items, although this tends to increase the internal consistency of the scale.

## Conclusion

In this study, the translation, cross-cultural adaptation, and evidence of validity based on the content and internal structure of an instrument that assesses pharmaceutical professionalism were presented. The investigation of its psychometric properties revealed a measure with six factors considered satisfactory for the professionalism of Brazilian pharmacists working in community pharmacies, hospitals, pharmaceutical clinics, public pharmacies, and pharmaceutical offices. This can effectively contribute to operationalize professionalism in places where this topic is poorly discussed or understood, being useful in future studies that consider its limitations. Besides, it can also assess the construct longitudinally, in sustainable and reproducible educational strategies.

### Supplementary Information

**Additional file 1.** 

**Additional file 2.** 

**Additional file 3.** 

**Additional file 4.** 

## Data Availability

Datasets used and/or analyzed during the current study made available by the corresponding author upon request.
