# Supplementary material for: Cross-cultural adaptation and psychometric evaluation of the “Modification of Hall’s professionalism scale for use with pharmacists”
Source: BMC Med Educ. 2023 Nov 16;23:871. doi: 10.1186/s12909-023-04815-y (PMC10655448; doi:10.1186/s12909-023-04815-y)
Supplement: Supplementary file 1 — Additional file 1. [file 12909_2023_4815_MOESM1_ESM.docx]

| **Criteria** | **Score** |
| --- | --- |
| Be a master in areas related to the sciences of professions | 2 Point |
| Be a master in areas related to health sciences with a dissertation in the areas of professions, professionalism or professional practice model | 1 Point |
| Being a doctor in areas related to the sciences of the professions | 1 Point |
| Being a doctor in areas related to health sciences with a thesis in the areas of professions, professionalism or professional practice model | 1 Point |
| Have surveys on model professions, professionalism or model professional practice | 2 Point |

ADDITIONAL FILE 1: Box: Fehring's criteria for selecting judges

ADDITIONAL FILE 1: Box: Characteristics of the experts who were part of the committee of judges

| **Sex** | **Age** | **Academic degree** | **Professional experience** |
| --- | --- | --- | --- |
| Female | 52 years | PhD | 26 years |
| Female | 47 years | PhD | 16 years |
| Male | 50 years | PhD | 20 years |
| Female | 52 years | PhD | 21 years |
| Female | 35 years | PhD | 9 years |
| Female | 63 years | PhD | 37 years |

ADDITIONAL FILE 1: Box: professionalism factors adopted in the construction of the instrument

| **Factor** | **Definition** |
| --- | --- |
| Autonomy | The professional believes that professional practice should not suffer lay judgments or interference, that is, from clients, people who are not members of their profession or legal interference. |
| Vocation | It reflects the professional's sense of personal commitment, pride and satisfaction with their work. |
| Professional council | Professional councils are the professional's main reference and reinforce ideals, beliefs, values and professional identity. They also dictate and influence standards of professional practice. |
| Self-regulation | The belief that only another professional from the same organization can judge the performance of another professional, so that there is no loss of control over one's own work. |
| Continuing education | The belief that the professional must continually develop knowledge, maintaining adequate levels of skills and competences. |
| Altruism | The professional's belief that their work is indispensable and brings benefits to society. Customer interests are above any other interest. |

Source: adapted from Schack and Hepler (1979)
