# Supplementary material for: Cross-cultural adaptation and psychometric evaluation of the “Modification of Hall’s professionalism scale for use with pharmacists”
Source: BMC Med Educ. 2023 Nov 16;23:871. doi: 10.1186/s12909-023-04815-y (PMC10655448; doi:10.1186/s12909-023-04815-y)
Supplement: Supplementary file 2 — Additional file 2. [file 12909_2023_4815_MOESM2_ESM.docx]

ADDITIONAL FILE 2: Comparison between the version before and after the evaluation by the target audience

| **Judges committee** | **Target audience assessment** |
| --- | --- |
| 1- My professional advice competently represents my views on pharmaceutical matters.  2- If I do not monitor the patient's pharmacotherapy, it is likely that the results will be unfavorable.  3- I think that we, pharmacists, should be the ones to determine and establish norms for our profession.  5- My employer should establish specific guidelines for making professional decisions in my work.  6- I can maintain an adequate standard of performance without having to participate in continuing education activities.  8- Patients would probably not be harmed if I failed to instruct them in the correct use of their medications.  10- No other profession makes me happier than Pharmacy.  12- Continuing education in the form of individual or group study (course, lectures, events) is essential for my professional practice.  14- It is impossible to achieve optimal pharmacotherapy for the patient without my services.  15- I would be willing to modify basic norms of my professional practice to adapt it to the wishes of the public.  16- Pharmaceutical practice is rewarding and satisfying for me.  18- My daily practice is the continuing education I need.  19- My professional advice provides a better understanding of the values and beliefs of the profession.  20- Patient care would be impaired without my services. | 1- My professional advice represents my views on pharmaceutical issues competently.  2- If I do not monitor the patient's pharmacotherapy, it is likely that his therapeutic results will be unfavorable.  3- I think that we, pharmacists, should be the only ones to determine norms related to attributions and competences of our profession.  5- My employer should establish specific guidelines for making professional decisions in my work.  6- I can maintain an adequate standard of performance without having to participate in continuing education activities (courses, lectures, events).  8- Patients would probably not be harmed if I failed to advise them on the correct use of their medications.  10- There is no profession in which I could be happier than Pharmacy.    12- Continuing education (courses, lectures, events) is essential for my professional performance.  14- It is impossible to achieve optimal pharmacotherapy for the patient without my clinical services.  15- I would be willing to change the basic norms of my professional activity to adapt it to the wishes of the public.  16- Pharmaceutical work is rewarding and satisfying for me.  18- My daily work is the continuing education (courses, lectures, events) that I need.  19- My professional advice gives me a better understanding of the values and beliefs of the profession.  20- Patient care would be impaired without my clinical services.  24- I would participate in continuing education activities (courses, lectures, events) only if they were necessary for the renewal of my professional registration.  25- The norms established by my professional council are important guides for my performance.  26- Patient care would be little impaired if I did not inform the doctor about pharmacotherapy.  28- I dedicate myself to the pharmaceutical profession because I believe in my work.  31- These are the professional norms that guide my work.  32- Continuing education (courses, lectures, events) has little importance for my professional performance.  33- I consider the pharmaceutical performance promoted by my professional council close to my expectations  34- Without my services, patient care would be unsatisfactory.  35- The population could contribute to the development of professional norms that guide my work.  38- My professional performance would be harmed if I did not participate in continuing education activities (courses, lectures, events)  39- There is better adherence to pharmacotherapy when I guide patients about the use of medication.  40- I would only modify professional norms after suggestions from pharmacists. |
| 24- I would participate in continuing education activities only if they were necessary for the renewal of my professional registration.  25- The standards established by my professional council are important guides for my practice.  26- Patient care would be little impaired if I did not inform the doctor about medications.  28- I dedicate myself to Pharmacy because I believe in my work.  31- These are the professional norms that guide my practice.  32- Continuing education has little importance for my professional practice.  33- I consider the pharmaceutical practice promoted by my professional council close to my ideal.  34- Without my services, patient care would be unsatisfactory.  35- The population could contribute to the development of professional norms that guide my practice.  38- My professional practice would be harmed if I did not participate in continuing education activities.  39- When I guide patients about the use of medication, adherence to pharmacotherapy improves.  40. I would change professional standards only in response to recommendations made by pharmacists. |  |
|  |  |
|  |  |
|  |  |
|  |  |
|  |  |
