# Supplementary material for: Cross-cultural adaptation and psychometric evaluation of the “Modification of Hall’s professionalism scale for use with pharmacists”
Source: BMC Med Educ. 2023 Nov 16;23:871. doi: 10.1186/s12909-023-04815-y (PMC10655448; doi:10.1186/s12909-023-04815-y)
Supplement: Supplementary file 3 — Additional file 3. [file 12909_2023_4815_MOESM3_ESM.docx]

ADDITIONAL FILE 3: Content Validity Coefficient of the items in the Brazilian version of the Modification of Hall’s Professionalism Scale for Use with Pharmacists

| ORIGINAL LANGUAGE | TRANSLATED VERSION | CL | PP | RT |
| --- | --- | --- | --- | --- |
| My professional organization competently represents my views on pharmacy issues. | My professional advice represents my views on pharmaceutical issues competently. | 0,77 | 0,73 | 0,70 |
| If i do not monitors patient drugs therapy, an unfavorable therapeutic outcome is probable. | If I do not monitor the patient's pharmacotherapy, it is likely that his therapeutic results will be unfavorable. | 0,87 | 0,80 | 0,83 |
| My pharmacy colleagues and i should be the only ones who determine and set standards for our practice. | I think that we, pharmacists, should be the only ones to determine norms related to the attributions and competences of our profession. | 0,83 | 0,97 | 0,93 |
| I often wish that i had chosen another occupation. | I often think that I should have chosen another profession. | 0,97 | 0,97 | 0,97 |
| My employer should establish specific guidelines for making professional decisions in my work. | My employer should establish specific guidelines for making professional decisions at work. | 0,87 | 0,83 | 0,87 |
| I can maintain an acceptable standard of practice without attending continuing education programs. | I can maintain an adequate standard of performance without having to participate in continuing education activities (courses, lectures, events). | 0,93 | 0,93 | 0,83 |
| My professional organization fails to promote advancement of the profession of pharmacy. | My professional advice fails to promote the advancement of the pharmaceutical profession. | 0,80 | 0,90 | 0,87 |
| Patients probably would not be harmed if i failed to instruct them concerning the proper use of their medications. | Probably, patients would not be harmed if I failed to advise them on the correct use of their medications. | 0,93 | 0,93 | 0,90 |
| The only professional standard i will accept are those established by my pharmacy colleagues. | The only professional norms I will accept are those set by us pharmacists. | 0,83 | 0,93 | 0,90 |
| There is no occupation i could be happier in than pharmacy. | There is no profession in which I could be happier than Pharmacy. | 0,83 | 0,83 | 0,70 |
| The opportunity to excise professional judgment in my work should be determined by my employer. | The judgment of my professional conduct must be determined by my employer. | 0,73 | 0,93 | 0,93 |
| Continuing education such as self-study or seminars is essential for my work. | Continuing education (courses, lectures, events) is essential for my professional performance. | 0,97 | 0,97 | 0,97 |
| My professional organization does not help to ensure quality practice. | My professional advice does not offer support to guarantee a quality performance. | 0,90 | 0,93 | 0,90 |
| Optimum drug therapy for the patient is impossible to achieve without my services. | It is impossible to achieve optimal pharmacotherapy for the patient without my clinical services. | 0,90 | 0,87 | 0,83 |
| I would be willing to modify the basic standards which guide my practice in order to conform to the wishes of the public. | I would be willing to modify basic norms of my professional activity to adapt it to the public's wishes. | 0,97 | 0,97 | 0,93 |
| The practice of pharmacy is gratifying and satisfying to me. | Pharmaceutical work is rewarding and satisfying for me. | 0,97 | 0,97 | 0,97 |
| My employer has the right to review and change the professional decisions i make. | My employer has the right to review and change my professional decisions. | 0,93 | 0,97 | 0,93 |
| My daily practice is all the continuing education i need. | My daily work is the continuing education (courses, lectures, events) that I need. | 0,70 | 0,87 | 0,83 |

*CL= language clarity; PP= practical relevance; RT= theoretical relevance Source: elaborated by the author

Table 1: Content Validity Coefficient of the items in the Brazilian version of the Modification of Hall’s Professionalism Scale for Use with Pharmacists (continued)

| ORIGINAL LANGUAGE | TRANSLATED VERSION | CL | PP | RT |
| --- | --- | --- | --- | --- |
| My professional organization provides me with a better understanding of the values and beliefs of my profession. | My professional advice gives me a better understanding of the values and beliefs of the profession. | 0,87 | 0,93 | 0,93 |
| The health care of the patient would suffer without my services. | Patient care would suffer without my clinical services. | 0,97 | 0,97 | 0,93 |
| Only another pharmacist is qualified to judge the competence of my work. | Only another pharmacist is qualified to judge the competence of my work. | 0,93 | 0,90 | 0,87 |
| If i had the opportunity to begin over again, i would choose top practice pharmacy. | If I had the opportunity to start over, I would still choose to be a pharmacist. | 0,93 | 0,93 | 0,93 |
| I would depart form my employers polices when i judge it professionally necessary. | I would disobey my employer's policies when I deemed it professionally necessary. | 0,67 | 0,97 | 0,97 |
| I would attend continuing education seminars only if they were required for prelicensure. | I would participate in continuing education activities (courses, lectures, events) only if they were necessary for the renewal of my professional registration. | 0,97 | 0,97 | 0,97 |
| The official statements and standards of my professional organization are important guides to my practice. | The norms established by my professional council are important guides for my performance. | 0,93 | 0,93 | 0,93 |
| Patient care would suffer very little if i failed to provide drug information to the physician. | Patient care would suffer little if I did not inform the doctor about drug therapy. | 0,80 | 0,80 | 0,80 |
| Pharmacists who violate professional standards should be judged only by this pharmacy colleagues. | Pharmacists who fail to comply with professional standards should be judged only by pharmacists. | 0,93 | 0,97 | 0,93 |
| I fell dedicated to pharmacy because i believe in my work. | I dedicate myself to the pharmaceutical profession because I believe in my work. | 0,97 | 0,97 | 0,97 |
| My employer has the right to influence my professional decision because my employer is the one who pays my salary. | My employer has the right to influence my professional decisions because he pays my salary. | 0,97 | 0,97 | 0,97 |
| My involvement with drug therapy has little consequence on prevention of adverse drugs reactions to the patient. | My involvement with the patient's pharmacotherapy has little bearing on preventing adverse drug reactions. | 0,90 | 0,93 | 0,90 |
| Standards for professional competence which guide my practice. | These are the professional norms that guide my work. | 0,93 | 0,97 | 0,97 |
| Continuing education is of little importance to my practice. | Continuing education (courses, lectures, events) has little importance for my professional performance. | 0,93 | 0,97 | 0,97 |
| The practice of pharmacy promoted by my professional organization is close to my personal ideal. | I consider the pharmaceutical performance promoted by my professional council close to my ideal. | 0,80 | 0,93 | 0,93 |
| Patient care would be unsatisfactory without my services. | Without my clinical services, patient care would be unsatisfactory. | 0,97 | 0,90 | 0,87 |
| The public should be allowed input into the development of standards for professional competence which guide my practice. | The population could contribute to the development of professional norms that guide my work. | 0,93 | 0,90 | 0,90 |
| I want other to enter pharmacy because i am proud of the unique skills and knowledge they would acquire. | I would like other people to study Pharmacy because I am proud of the unique skills and knowledge they can acquire. | 0,90 | 0,90 | 0,90 |

*CL= language clarity; PP= practical relevance; RT= theoretical relevance Source: elaborated by the author

Table 2: Content Validity Coefficient of items from the Brazilian version of the Modification of Hall’s Professionalism Scale for Use with Pharmacists.

| ORIGINAL LANGUAGE | TRANSLATED VERSION | CL | PP | RT |
| --- | --- | --- | --- | --- |
| My employer has no right to place limitations on the decisions i make concerning professional matters. | My employer does not have the right to limit the decisions I make about professional matters. | 0,90 | 0,90 | 0,90 |
| My practice would suffer if i did not attend continuing education programs. | My professional performance would be impaired if I did not participate in continuing education activities (courses, lectures, events) | 0,93 | 0,90 | 0,90 |
| Patient drugs compliance is improved by my explanation of drug therapy to patients. | There is better adherence to pharmacotherapy when I guide patients about the use of medications. | 0,97 | 0,97 | 0,97 |
| I would modify the professional standards which guide my practice only in response to recommendations made by my pharmacy colleagues. | I would only modify professional norms after suggestions from pharmacists. | 0,97 | 0,93 | 0,90 |

*CL= language clarity; PP= practical relevance; RT= theoretical relevance Source: elaborated by the author
