# Supplementary material for: Cross-cultural adaptation and psychometric evaluation of the “Modification of Hall’s professionalism scale for use with pharmacists”
Source: BMC Med Educ. 2023 Nov 16;23:871. doi: 10.1186/s12909-023-04815-y (PMC10655448; doi:10.1186/s12909-023-04815-y)
Supplement: Supplementary file 4 — Additional file 4. [file 12909_2023_4815_MOESM4_ESM.docx]

ADDITIONAL FILE 4: Brazilian version of the Modification of Hall’s Professionalism Scale for Use with Pharmacists

| ITEM |
| --- |
|  |
| 1: My professional advice represents my views on pharmaceutical issues competently. |
| 2: If I do not monitor the patient's pharmacotherapy, it is likely that his therapeutic results will be unfavorable. |
| 3: I think that we, pharmacists, should be the only ones to determine norms related to the attributions and competences of our profession. |
| 4: I often think that I should have chosen another profession. |
| 5: My employer should establish specific guidelines for making professional decisions in my work. |
| 6: I can maintain an adequate standard of performance without having to participate in continuing education activities (courses, lectures, events). |
| 7: My professional advice fails to advance the pharmaceutical profession. |
| 8: Patients would probably not be harmed if I failed to advise them on the correct use of their medications. |
| 9: The only professional norms I will accept are those set by us pharmacists. |
| 10: There is no profession in which I could be happier than Pharmacy. |
| 11: The judgment of my professional conduct must be determined by my employer. |
| 12: Continuing education (courses, lectures, events) is essential for my professional performance. |
| 13: My professional advice does not offer support to guarantee a quality performance. |
| 14: It is impossible to achieve optimal pharmacotherapy for the patient without my clinical services. |
| 15. I would be willing to modify basic norms of my professional performance to adapt it to the public's wishes. |
| 16. Pharmaceutical work is rewarding and satisfying for me. |
| 17. My employer has the right to review and change my professional decisions. |
| 19. My professional advice gives me a better understanding of the values and beliefs of the profession. |
| 20. Patient care would suffer without my clinical services. |
| 21. Only another pharmacist is qualified to judge the competence of my work. |
| 22. If I had the opportunity to start over, I would still choose to be a pharmacist. |
| 23. I would disobey my employer's policies when I deemed it professionally necessary. |
| 24. I would participate in continuing education activities (courses, lectures, events) only if they were necessary for the renewal of my professional registration. |
| 25. The standards established by my professional council are important guides for my performance. |
| 26. Patient care would suffer little if I did not inform the doctor about drug therapy. |
| 27. Pharmacists who fail to comply with professional standards should be judged only by pharmacists. |
| 28. I dedicate myself to the pharmaceutical profession because I believe in my work. |
| 29. My employer has the right to influence my career decisions because he pays my salary. |
| 30. My involvement with the patient's pharmacotherapy has little influence on preventing adverse drug reactions. |
| 31. Professional norms guide my work. |
| 32. Continuing education (courses, lectures, events) has little importance for my professional performance. |
| 33. I consider the pharmaceutical activity promoted by my professional council close to my ideal. |
| 34. Without my clinical services, patient care would be unsatisfactory. |
| 35. The population could contribute to the development of professional norms that guide my work. |
| 36. I would like other people to study Pharmacy because I am proud of the unique skills and knowledge they can acquire. |
| 37. My employer does not have the right to limit the decisions I make about professional matters. |
| 38. My professional performance would be harmed if I did not participate in continuing education activities (courses, lectures, events). |
| 39. There is better adherence to pharmacotherapy when I advise patients on the use of medication. |
| 40. I would only change professional standards after suggestions from pharmacists. |
